# A ten-year retrospective analysis of medical school rankings in the United Kingdom

**DOI:** 10.1186/s12909-025-08538-0

**Published:** 2026-01-21

**Authors:** Owen W. Tomlinson, James Butler

**Affiliations:** Department of Clinical and Biomedical Sciences, University of Exeter Medical School, Exeter, EX1 2LU UK

**Keywords:** Rankings, Metrics, Prestige, Selection, Recruitment, Medicine

## Abstract

**Background:**

National rankings are employed by higher education (HE) institutions to guide strategic decisions and by prospective applicants and their supporters to guide decisions about where to study. Given the increased marketisation of HE and the use of rankings in decision making, it is essential to understand the nuances of rankings: how different systems lead to different rankings, the correlation between rankings of high-profile programmes such as medicine and that of their institutions, and how rankings shift over time. To date, no studies have assessed agreement between ranking systems for medical programmes in the United Kingdom (UK). Given that various ranking systems are used globally to compare institutions within and across countries, a deeper understanding of these ranking systems is crucial.

**Method:**

Retrospective data across a ten-year period (2016–2025) were obtained on the national rankings of medicine programmes in the UK: the Complete University Guide, Guardian, and The Times. The data were analysed to investigate agreement in ranking of medical programmes over time and the agreement between overall ranking of an institution and the ranking of its medical programme.

**Results:**

Ranking systems showed a high level of agreement with each other. However, mean scores over the ten-year period varied by ranking system, whereby institutions occupying the ‘top three’ positions were not wholly consistent across systems. Associations between institutional rank and that of institutions’ medicine programmes were statistically significant (*r* = 0.36–0.38, *p* < 0.01).

**Conclusions:**

Agreement between ranking systems for medical programmes in the UK is present, yet there remains notable variation for some institutions within those ranking systems, likely due to different components and weightings that make up these scores. The weak association between overall rank and medical ranks implies that the presence of a medical school at an institution does not guarantee a high overall rank – higher universities can have lower medical schools, and vice versa. This suggests that prospective students should consider an array of factors when deciding on where to study; not rankings alone.

## Background

In medical education, as in higher education (HE) more broadly, institutions are ranked by various bodies in an effort to quantify and compare their teaching and research quality, prestige, graduate outcomes and student experience. In the competitive field of medical applications, rankings have the potential to influence prospective applicants and the supporters of those applicants as a measure of prestige guiding decision making. In an increasingly marketised higher education system [1, 2], rankings carry weight, affecting the behaviour of universities, governments, funding agencies, academics and students, and are even reportedly used to make decisions on immigration in some jurisdictions [3].

Specifically, in the UK, medical schools are ranked by three main systems: *Complete University Guide*, *The Guardian*, and *The Times*. Information sources include the Office for Students (OfS) and Higher Education Statistics Agency (HESA), themselves taking data from the National Student Survey (NSS) and Research Excellence Framework (REF). There are 41 institutions with awarding powers and regulatory compliance for primary medical qualifications in the UK, as recognised by the General Medical Council [4]. Although, it should be noted that not all 41 institutions are always included in rankings; potentially due to the availability of data or due to newer medical schools not yet producing the outputs being compared.

*Complete University Guide* reports [5] that it ranks universities based on entry standards (average UCAS tariff score), student satisfaction (NSS), research quality and intensity (REF), graduate prospects (Graduate Outcomes Survey, HESA), student-staff ratio, academic services and facilities spend, and continuation rate (HESA). *The Guardian* reports [6] that it ranks universities based on entry standards (average UCAS tariff score), student-staff ratios, expenditure per students, continuation, student satisfaction (NSS), “value added”, and career prospects. *The Times* reports [7] that it ranks universities using data on entry standards, student-staff ratios, continuation rates, level of award (first-class and 2:1), student satisfaction (comprising teaching quality and student experience), research quality (REF), ‘people and planet’ (14 ethical and environmental criteria) and graduate prospects. However, across the board, graduate prospects scores and levels of awards do not inform rankings for medicine. Generally, across ranking systems, different factors are weighted differently depending on subject, leading to variances in where institutions sit within each ranking system.

Ranking of institutions is a global phenomenon not confined to the UK. For example, in the US, annual medical school rankings have been the subject of sustained criticism [8, 9] and many of the most prestigious medical schools have withdrawn from national ranking systems such as the *U.S. News & World Report* [10]. Criticism has been based on purported flawed methods and underlying conceptual flaws [8]. There are some clear areas of difference between the sociocultural contexts in the US and UK, for example in the US, medical schools may be categorised based on their secular versus religious heritage [8] while in the UK all medical schools would be considered to be secular. This is an example of a sociocultural factor that would affect decisions regarding where to study in the US but not in the UK. However, despite unavoidable differences when comparing international education systems, there are clearly familiar characteristics such as the institutional focus on rankings as a key performance indicator, and the high regard with which rankings are held by the public and the profession – described in the US as a “cultural obsession with selectivity, status, and elitism” [9].

Critically, within the UK, *Times Higher Education* lists seven reasons to participate in the World University Rankings: (1) organisational management and strategy, (2) brand and visibility, (3) collaboration and partnerships, (4) recruitment, (5) benchmarking and analysis, (6) data collection, and (7) reputation [11]. There are some instances of institutions withdrawing or choosing not to be included in UK university rankings. For example, Birkbeck, University of London opts out due to its focus on part-time HE. The Open University, which offers distance learning, is not considered to be fairly comparable. Most recently, the University of the Highlands and Islands has withdrawn from *The Times* rankings, “owing to its unique structure over 70 sites and a large number of part-time staff” [7]. However, none of these institutions offers medical programmes.

Some authors have addressed the correlation between a medical school’s ranking and metrics such as their graduates’ specialty choice [12] and attainment of national awards [13]. In the US, it was found that students in top-ranked medical schools were less likely to be influenced by factors such as length of training or work-life balance and were also less likely to be interested in pursuing specialisation in primary care [12]. This was considered to be due to both selection and education of the students concerned. Selection is a significant factor, with UK evidence suggesting that students from state school backgrounds tend to do better in certain post-graduation outcome measures despite doing less well by admissions/selection measures [14] as compared to students from fee-paying school backgrounds. In a UK study, it was found that the majority (60.4%) of pathologists who were national merit award-winners originated from only five UK medical schools [13]. A similar study found that 56.4% of all UK anaesthetist national award winners had attended the ‘top’, albeit different, five medical schools [15]. Selection is also significant due to the ‘Matthew effect’, which can be summarised as ‘advantage begets advantage’ or the ‘principle of cumulative advantage’ [16] – top ranked medical schools are likely to attract the highest performing applicants [17], leading to a cycle of producing high quality graduates, which potentially perpetuates the high ranking. Further investigation would be needed to understand the nuance of how this effect fits together with the finding above regarding state school students’ performance in outcome measures.

The landmark study in the analysis of how medical schools compare was the 2020 ‘*MedDifs*’ study which investigated selection, teaching, students and foundation doctor (F1) perceptions, postgraduate outcomes and fitness to practise [18]. Whilst this study found that medical schools differ in many ways that are “causally interconnected”, the study described medical school differences as stable across time. However, this study did not address or investigate rankings of medical schools. It is unknown how well ranking systems in the UK agree with one another, and therefore whether an undue influence upon student decisions on where to apply may be conferred by one particular system. It is also unknown how well rankings of medical programmes align with the overall profile of an institution. This article analyses a decade of retrospective data to address these gaps in understanding of medical school rankings.

## Methods

Retrospective data from 2016 to 2025, a period spanning ten consecutive years, was obtained on the national ranking of institutions’ medicine programmes in the UK. These were obtained for each of the *Complete University Guide*, *The Guardian*, and *The Times*. All data from ranking organisations are available either on host websites, or via published guides, and therefore no proprietary information that would otherwise not be in the public domain is published here.

Firstly, Spearman’s rank correlation coefficients established agreement in ranking of medical programmes between ranking systems over a cumulative ten-year period, and secondly, mean scores for each institutional medical programme were ranked over a ten-year period per system. These were undertaken per ranking system, and as a cumulative whole.

Finally, agreement between overall ranking of an institution, and the ranking of its medical programme was assessed by Spearman’s coefficients. This was undertaken within each system in individual years and over a cumulative ten-year period.

Due to variances in ranking systems, not all institutions are included in each year’s ranking for each system, and only institutions that explicitly run accredited medical programmes were included (ancillary programmes such as medical science and biomedicine are not included). Data are only analysed when provided, and no imputation is undertaken. For all rankings, a score of ‘1’ is considered best, and ‘2’ is second best, and so on. When ranking programmes, if institutions have equal mean rank, then those with a smaller standard deviation (SD) are ranked higher, indicating less variance in their positioning (i.e. more consistency). For all analyses, statistical significance was set at *p* < 0.05.

## Results

**Fig. 1:**
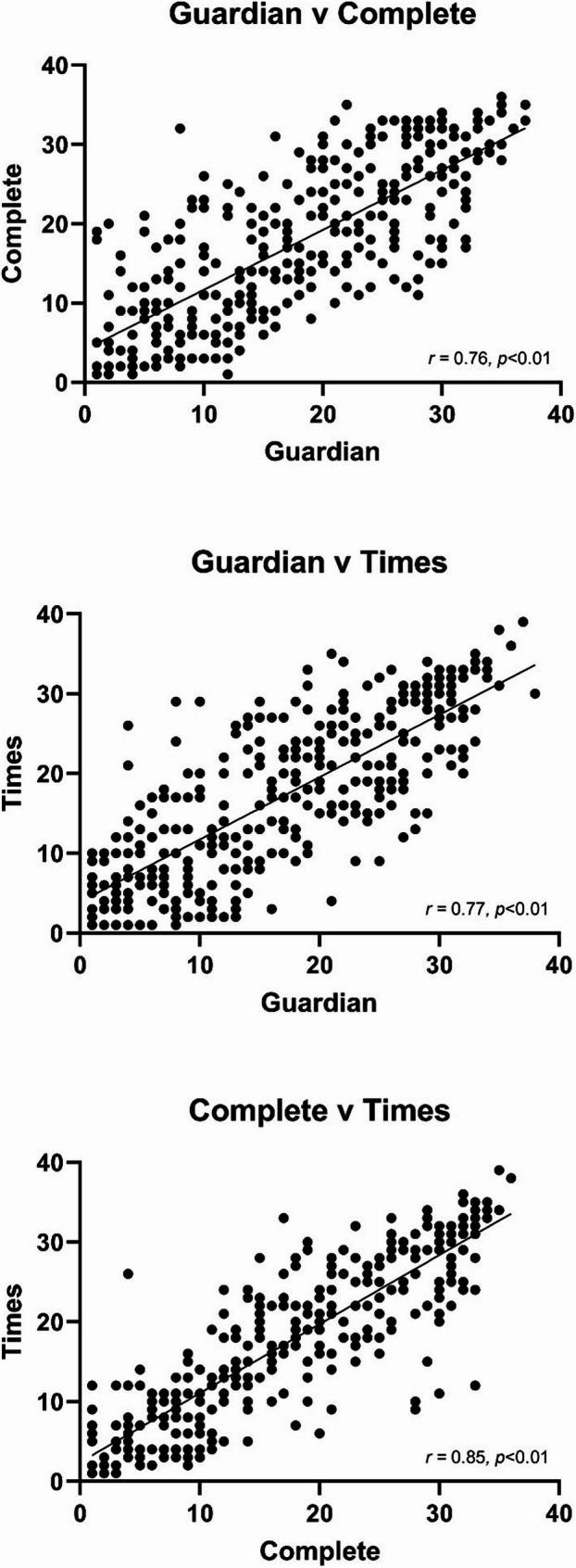
Agreement between ranking systems for medical programmes over a cumulative ten-year period

Ranking systems displayed a high level of agreement between one another, when cumulative ranks of medical programmes were considered over the ten-year period. The *Complete University Guide* and *The Times* showed the highest agreement (*r* = 0.85, *p* < 0.01; Fig. 1).

**Fig. 2:**
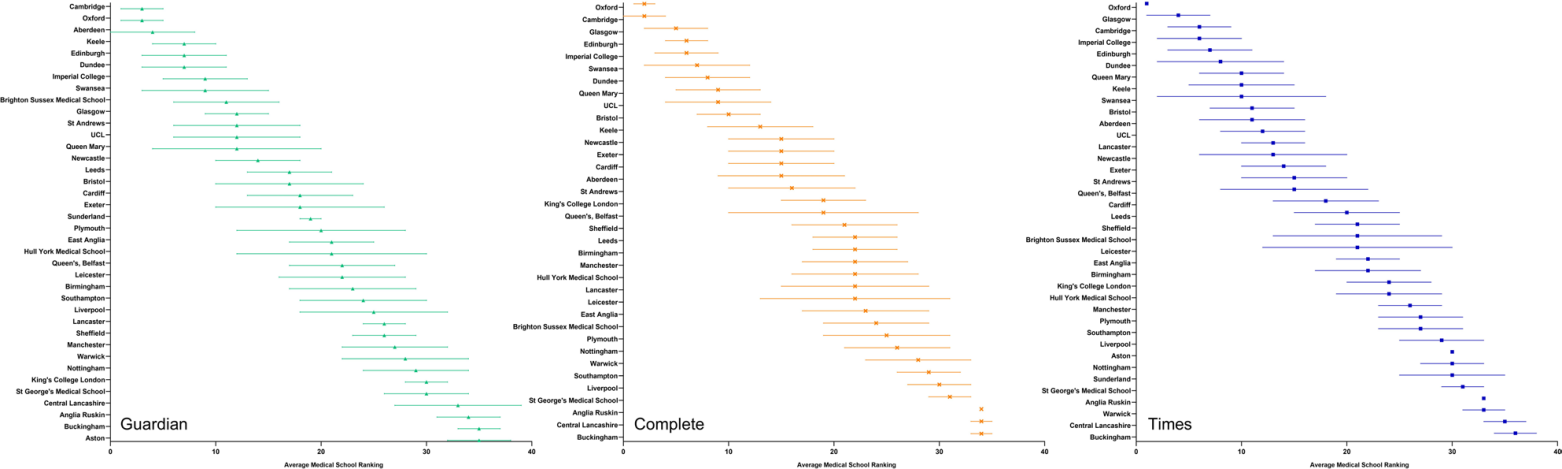
Mean rankings of medical programmes per institution over a ten-year period from 2016–2025, across each ranking system. Scores are displayed as mean ± standard deviation. When institutions have equal mean rank, the institution with the smallest standard deviation is ranked higher

**Fig. 3:**
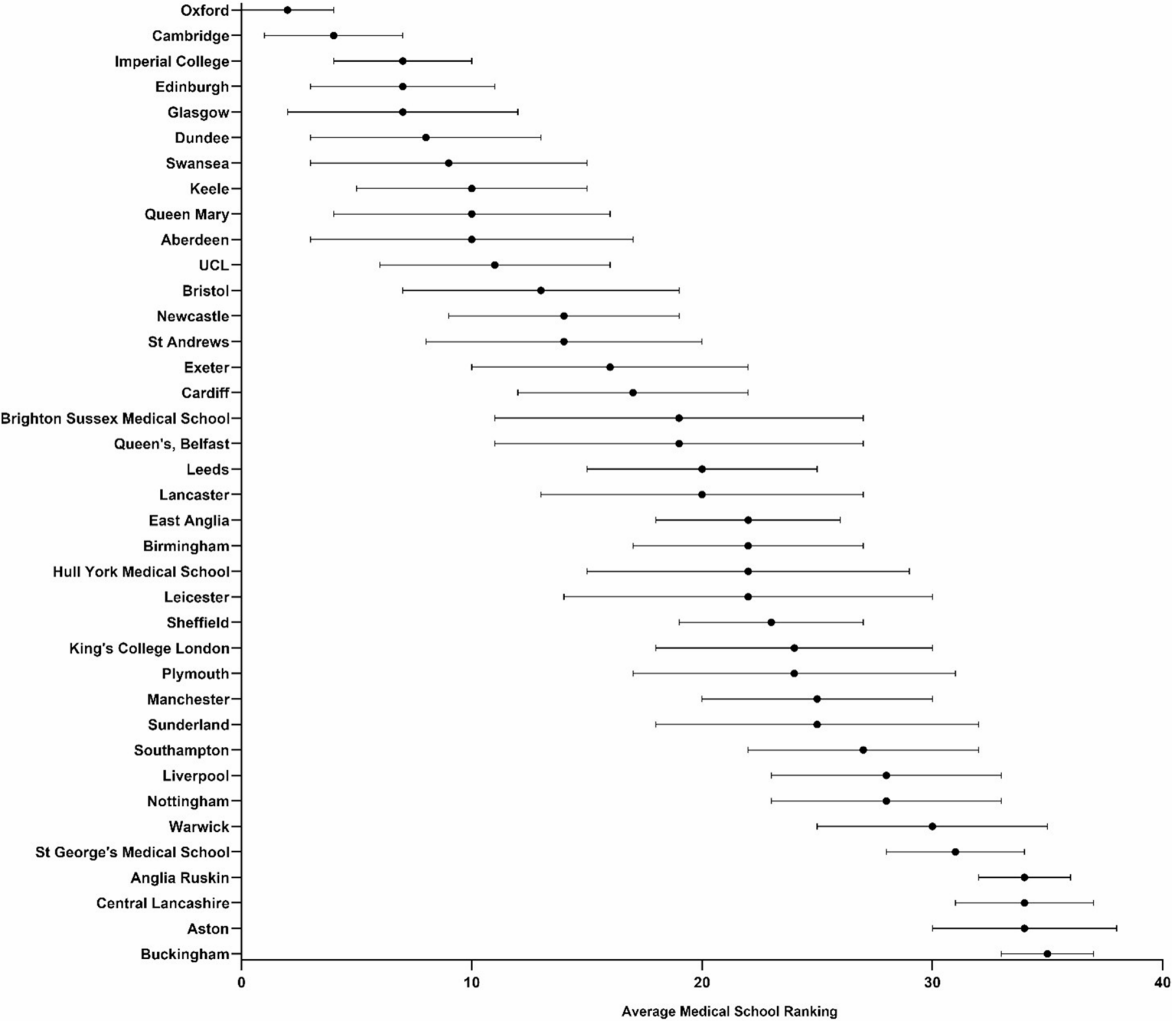
Mean ranking of medical programmes over a ten-year period, averaged across all ranking systems. Scores are displayed as mean ± standard deviation. When institutions have equal mean rank, the institution with the smallest standard deviation is ranked higher

Mean scores for individual institutions over a ten-year period varied by ranking system. For example, according to *The Times* and the *Complete University Guide* systems, the programmes offered by Oxford, Cambridge, and Glasgow consistently had the three highest mean scores, albeit in differing orders. In contrast, as per *The Guardian*, programmes offered by Oxford, Cambridge, and Aberdeen consistently ranked the highest over the same ten-year period (Fig. 2). When all three systems are subsequently averaged over the ten-year period, programmes offered by Oxford (mean rank 2 ± 2), Cambridge (4 ± 3), Imperial College London (7 ± 3), Edinburgh (7 ± 4), and Glasgow (7 ± 5) consistently ranked highest (Fig. 3).

**Fig. 4:**
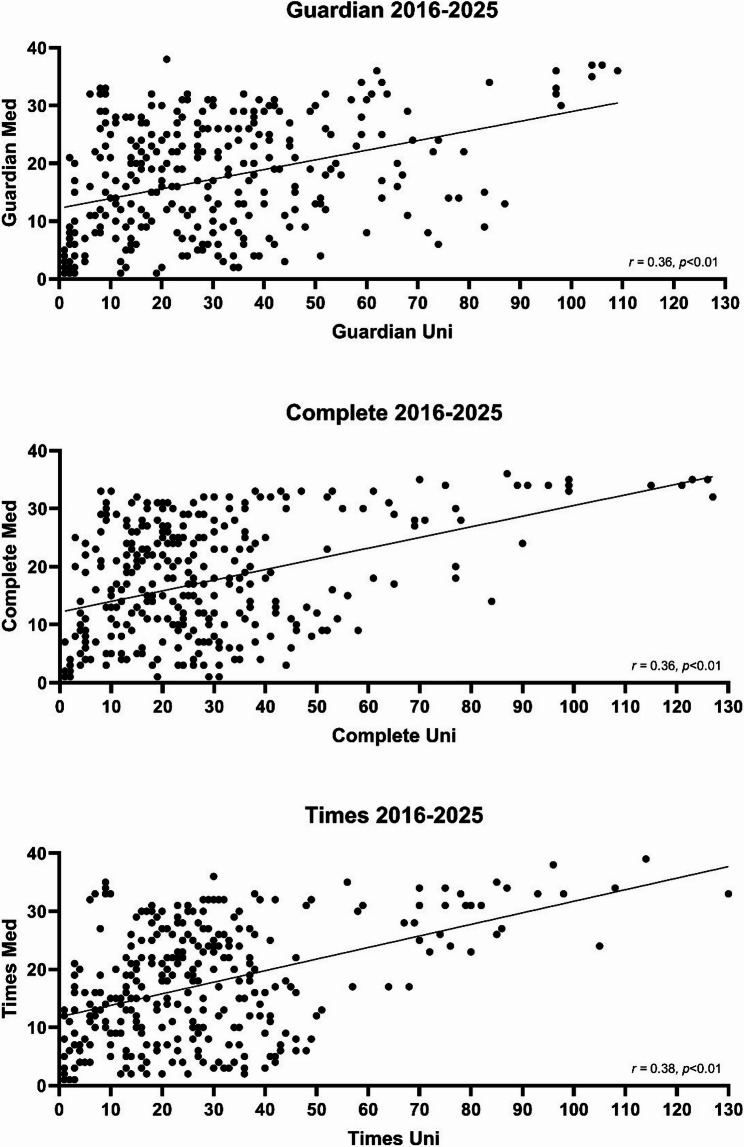
Correlation between overall institutional rank, and rank of medical programme

Finally, when considering overall institutional rank and ranking of medicine programmes, statistically significant associations are identified for each ranking system, but these are weak correlations (Fig. 4).

## Discussion

In this article, we have explored a decade of retrospective UK medical school ranking data with a view to understand trends and, ultimately, provide indications of the utility of such a ranking system for prospective students, institutions and other stakeholders.

On the surface, the ranking systems of *The Guardian*, *Complete University Guide* and *The Times* tend to agree, as shown by correlation coefficient values of 0.76–0.85 and *p* < 0.01 (Fig. 1). This is to say that medical programmes highly ranked by one system are likely to be highly ranked by the others. However, there is also a level of inconsistency between and within ranking systems (Fig. 2). As selected examples, Aberdeen and Brighton-Sussex are well ranked by *The Guardian* (averaging #3 and #9 respectively), however, Aberdeen is mid-table for *Complete University Guide* (averaging #15), with Brighton-Sussex ranked much lower (averaging #27). Within systems, some institutions have high variance, implying lots of movement (e.g. *The Guardian*, Hull York; *Complete University Guide*; Queens Belfast; *The Times*, Leicester).

The level of variance and closely overlapping standard deviations could be interpreted to imply that the different weighting of metrics across systems can have a significant influence. However, this would not explain the level of variance seen in rankings over the ten-year period by a given single methodology (Fig. 2). This would seem to suggest a ‘tight pack’ of medical schools with small differences that lead to adjustments in rankings from year to year. Variation of rankings have far-reaching consequences for institutions, with evidence suggesting that those identified as more prestigious are more significantly affected by ranking changes with reductions in both average tariff of prospective applicants, total applicant numbers, and numbers of EU and international applicants [17].

In Fig. 3, we have compiled a mean ranking of medical programmes over the past decade. This analysis distils Oxford and Cambridge as consistent first and second place, respectively, with minimal variance across the majority of medical schools’ rankings. There appears to be a trend of older, more established medical schools ranking higher over the decade whilst the newer institutions, such as Aston, Central Lancashire and Anglia Ruskin, ranked lower. Interestingly, six of the top 10, and thirteen of the top 20 institutions are members of the research-intensive Russell Group: around a two-thirds ‘dominance’ for a national group of 24 institutions. There is indeed evidence to suggest that newer medical schools do less well than more established medical schools over different metrics [18]. This begs the question of whether newer institutions will climb the rankings and displace some of the more established, but potentially poorer performing, institutions. This is yet to be seen, and observation over the coming decades will be needed.

It is interesting to see that whilst institutional rank does correlate significantly with medicine subject rank (*p* < 0.01), this correlation is weak with coefficient values of 0.36–0.38. This finding could be interpreted as refuting what we consider, anecdotally, to be the common notion that universities with a medical school are by definition high quality, or that a well-ranked institution will inevitably establish a well-ranked medical programme. This would suggest that prospective students should consider a university as a whole when applying to medical school, as the presence of a medical school itself can be misleading and some ‘lower’ ranked institutions can be just as good, if not better, when it comes to their medical programme. With the wide range of different teaching styles and curriculum designs across medical schools, for example integrated versus traditional, and lecture-based versus small group learning based, prospective students should make informed decisions based on the teaching style and environment that best suits their preference and is consequently more likely to foster success. Figure 3 ultimately shows large variance in medical school rankings amongst the majority of the list, and it is likely that small differences in quality are being overblown by a system that forces institutions to be placed higher or lower than others, without much nuance.

There are a number of complex confounding factors to consider regarding the decision-making process for prospective students. This makes it challenging to understand how rankings attract or deter students, and how this ultimately goes on to affect rankings. For example, newer medical schools tend to employ variations of problem-based learning (PBL) and there is evidence to suggest that graduates of PBL schools perform less well on postgraduate assessments than those from other institutions without PBL [18]. It is difficult however to understand if this assessment performance is due to PBL teaching and learning itself, differences in the standards of students attending PBL schools (due to reportedly lower entry grades [18]), or a combination of multiple factors. This effect, if indeed true beyond the evidence presented by McManus et al. [18], is not explainable by notions of institutional knowledge or experience as the sponsorship model of expansion ensures that established medical schools sponsor new ones. Furthermore, the activity of the General Medical Council, the UK regulator ensuring rigorous standards and quality [19], should ensure equivalence of medical degrees.

Within this analysis, there are several strengths, notably the use of ten years’ worth of data, resulting in > 350 data points for analysis. Moreover, our analysis can be replicated by others as we have made use of openly accessible data. In addition, by reporting on the same three ranking systems we have ensured internal validity of ranking process (regardless of how ‘good’ each system actually is), and those that are specific to the UK, which allocate explicit ranks to each institution. This is unlike international rankings (e.g. *Times Higher Education*, *QS World*, *Shanghai*) which all cluster categorical ranks after a certain threshold (e.g. 1,2,3…up to 99, followed by categorical ranks of 100–150, 151–200 etc.). These larger, international systems lack some nuance and therefore, in our focus of the UK ranking system we have brought UK-specific nuance. Finally, many, if not the vast majority of UK medical graduates will go into practice in the National Health Service (NHS) and will follow similar clinical training programmes in terms of placements. This ensures a level of parity in the clinical training component of programmes that are ranked, potentially avoiding confounding variables introduced by different clinical placements available in different institutions in countries without unified national healthcare.

In contrast, we acknowledge some limitations of our analysis. Firstly, the 10-year span of data covers periods of change in government policy around HE and health, as well as shifting trends in terms of prospective student demographics. For example, in this time period there have been five different Prime Ministers, seven Secretaries of State for Health and Social Care, eight Ministers of State for Education, and 10 Secretaries of State for Education. While there have not been major policy shifts in this time, for example tuition fees were raised to their current level of >£9,000 per year before the start of the decade being analysed, it is pertinent to consider a shifting HE landscape as a backdrop to HE rankings applied to medicine. Moreover, there is unavoidable internal cross-over between each system’s pool of data feeding into rankings; e.g. make use of NSS and/or REF but to different extents. The exact ranking methodologies in terms of differential weighting are also opaque and can present a challenge when trying to ‘tease apart’ differences.

## Conclusions

Our analysis of a decade of UK medical school rankings data has shown broad agreement between ranking systems over the ten-year period. There was some notable variation in mean ranks of institutions, leading to some differences in top positions depending on ranking system. To reiterate that top ranked medical programmes vary depending on which ranking system is being employed, averaging of ranks across systems and years lead to significant error in predicting rank due to overlap of data. Significant correlations between ranking of medical programmes and the university offering the programme were found, although these were weak, possibly suggesting only a modest link between well-ranked institutions and well-ranked medical schools. Given these findings, we would suggest that it would be prudent for applicants to consider factors such as their preferred pedagogical approach, the size and geographical location of the institution and medical programme, and their interest in involvement in research and intercalation.

## Data Availability

All data from ranking organisations are available either on host websites, or via published guides, and therefore no proprietary information that would otherwise not be in the public domain is published here.

## Abbreviations

HE: Higher Education
HESA: Higher Education Statistics Agency
NHS: National Health Service
NSS: National Student Survey
OfS: Office for Students
PBL: Problem–based learning
REF: Research Excellence Framework
SD: Standard deviation
UK: United Kingdom
US: United States
